# Multimodal Radiographic Diagnosis of a Complex Müllerian Anomaly: A Case Report

**DOI:** 10.7759/cureus.67967

**Published:** 2024-08-27

**Authors:** Arielle N Valdez-Sinon, Marika A Toscano, Valerie L Baker, James Segars, Jaden R Kohn

**Affiliations:** 1 Department of Gynecology and Obstetrics, Johns Hopkins University School of Medicine, Baltimore, USA; 2 Division of Maternal-Fetal Medicine, Department of Gynecology and Obstetrics, Johns Hopkins University School of Medicine, Baltimore, USA; 3 Division of Reproductive Endocrinology and Infertility, Department of Gynecology and Obstetrics, Johns Hopkins University School of Medicine, Baltimore, USA; 4 Division of Reproductive Science and Women's Health Research, Department of Gynecology and Obstetrics, Johns Hopkins University School of Medicine, Baltimore, USA; 5 Division of General Obstetrics and Gynecology, Department of Gynecology and Obstetrics, Johns Hopkins University School of Medicine, Baltimore, USA

**Keywords:** müllerian anomaly, uterine anomaly, radiography, infertility, vaginal septum, uterine didelphys

## Abstract

As evidenced by the 2021 American Society for Reproductive Medicine Müllerian Anomaly Classification (ASRM MAC), there are numerous possible configurations of the female genitourinary system. Some anomalies place patients at higher risk of infertility, miscarriage, fetal malpresentation, and preterm labor. Correct characterization of Müllerian anomalies is critical for proper infertility treatment and pregnancy counseling. This case study of a 32-year-old nulliparous woman describes the radiographic modalities utilized in the diagnosis of a complex Müllerian anomaly. To characterize the Müllerian anomaly, the patient underwent a 2D transvaginal ultrasound (TVUS), saline infusion sonohysterogram (SIS) with 3D reconstruction, and second-opinion interpretation of a previous MRI. Radiographic diagnoses were conflicting among different modalities. While the saline infusion sonohysterogram suggested a bicornuate uterus, the MRI of the anomaly was interpreted as a uterine didelphys. Furthermore, TVUS and MRI indicated the presence of two cervices, while only one cervix was appreciated with SIS. Given the inconsistent interpretation of her anomaly, the patient was brought to the operating room. After direct visualization with exam and hysteroscopy, the patient was ultimately diagnosed with a uterine didelphys with a communication of endometrial cavities in the lower uterine segment, two cervices, a hypoplastic right vagina, and a longitudinal vaginal septum extending to the hymen. The patient was counseled that this anomaly is not expected to impact natural conception and would not require surgical resection. The patient went on to conceive spontaneously in the right uterine horn, with a plan for primary cesarean delivery. This case was of sufficient complexity that an accurate diagnosis was not made until physical exam and hysteroscopic visualization verified findings from MRI and ultrasonographic imaging. Appropriately describing a Müllerian anomaly according to the 2021 ASRM MAC is crucial, and multiple forms of imaging and physical examination may be necessary to accurately characterize uterine anomalies.

## Introduction

The 2021 American Society for Reproductive Medicine Müllerian Anomaly Classification (ASRM MAC) demonstrates a broad range of complexities amongst the anatomic variants [1]. Just as there is diversity within the types of Müllerian anomalies, presenting symptoms may vary. While many patients are asymptomatic in the presence of Müllerian anomalies, obstructive anomalies may cause symptoms at the time of menstruation [2]. Anomalies with a complete obstruction such as cervical agenesis or a transverse vaginal symptom may lead to primary amenorrhea with cyclic pelvic pain [2]. Anomalies with partial obstruction such as a noncommunicating uterine horn may also cause cyclic pelvic pain. Vaginal anomalies (i.e., vaginal symptoms) may present with dyspareunia or difficulty with tampon insertion. Recurrent pregnancy loss may be the primary symptom that patients with uterine anomalies (i.e., septate uteri and arcuate uteri) experience [3] that prompt them to undergo evaluation.

Despite similarities shared amongst some Müllerian anomalies, the impacts on fertility and pregnancy are varied [4,5]. For example, a septate uterus is most associated with recurrent pregnancy loss (6-16% of cases), whereas unicornuate, arcuate, and bicornuate uteri are reported in only 0.5-2% of recurrent pregnancy loss cases [3]. The prevalence of Müllerian anomalies in patients with a history of infertility and miscarriage can be as high as 25% [5]. Thus, in patients with infertility or recurrent pregnancy loss, evaluation for uterine structural anomalies (including potential Müllerian anomalies) is important. Accurate diagnosis of an anomaly is essential for proper patient counseling and discussion of management options. While MRI is the gold standard for the evaluation of Müllerian anomalies [6], others have proposed that more cost-effective imaging modalities such as 3D ultrasound have comparable accuracy for diagnosis with less risk and cost [7]. Presented below is a case of a patient with varying radiographic interpretations of a Müllerian anomaly who was subsequently diagnosed conclusively according to the 2021 ASRM MAC with an exam under anesthesia and hysteroscopy. 

## Case presentation

**Figure 1:**
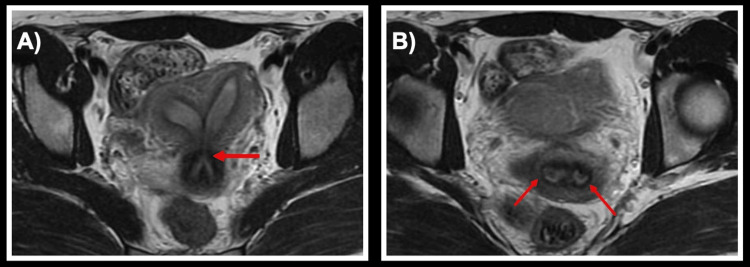
MRI demonstrating uterine didelphys and cervix bicollis (A) Two distinct uterine cavities with an area of communication (indicated by an arrow). (B) Two distinct cervical canals (each canal indicated by an arrow).

The patient was a 32-year-old nulliparous woman who initially sought gynecologic care at an outside hospital for dysmenorrhea and menorrhagia that had been persistent since the onset of menarche at age 13. A pelvic exam at the time of initial presentation was notable for one midline normal cervix with a possible rudimentary cervix near the vaginal fornix, and no vaginal septum was visualized. Transvaginal ultrasound (TVUS) noted two separate endometrial cavities with possibly two separate cervices, concerning for bicornuate uterus versus uterine didelphys. The incongruence between her pelvic exam and TVUS further made the diagnosis unclear. A follow-up MRI from the same outside institution (Figure 1) interpreted her anomaly as a septate uterus with a muscular upper septum and avascular lower septum that appeared to extend into the upper vagina, along with two cervices. MRI interpretation characterized the fundal contour of the uterus as flat. Bilateral adnexa and renal structures were normal on both ultrasound and MRI imaging. Since septate uteri are associated with impaired fertility outcomes compared with uterine didelphys [4] and uterine septa may require surgical resection, the varying interpretations of her imaging and physical exam findings had different implications for her fertility planning. 

**Figure 2:**
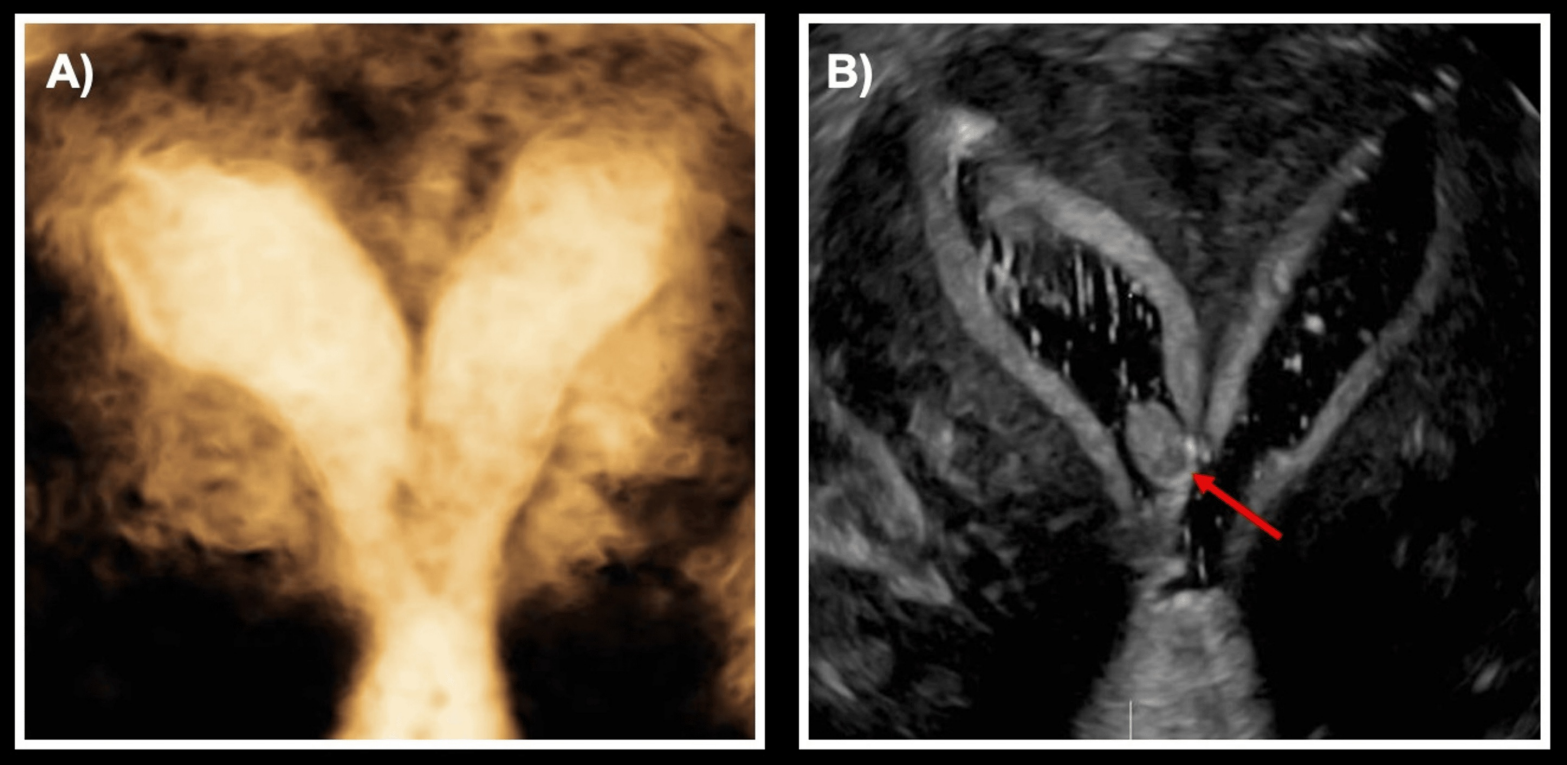
Saline infusion sonohysterogram with 3D reconstruction (A) 3D reconstruction demonstrating two distinct horns and a connection in the lower uterine segment. (B) Endometrial polyp in the right uterine cavity (indicated by an arrow).

When the patient was planning to conceive a few years after her initial work-up, she then presented for preconception counseling to this institution in the setting of her unclear Müllerian anomaly. She continued to have dysmenorrhea and abnormal uterine bleeding. Given the conflicting results from her previous imaging and physical exam, she was recommended for a saline infusion sonohysterogram (SIS) for further delineation of the intrauterine cavity. Given the patient's history of dyspareunia, a thorough pelvic examination at the time of SIS was difficult; a vaginal septum and hemi-vagina could not be appreciated. Only one cervix could be visualized and cannulated during the SIS. SIS with 3D reconstruction demonstrated a single cervical canal and uterus divided into two communicating horns with a concave fundal contour - most consistent with a bicornuate uterus (Figure 2). A small filling defect was noted in the lower segment of the right horn, suggesting an endometrial polyp versus submucosal fibroid. The speculum exam at the time of SIS demonstrated a single vagina and single cervix without a vaginal septum. 

Given the three varying interpretations of the Müllerian anomaly with multiple imaging studies, the MRI from the outside hospital was reviewed for a second opinion and then discussed during a standing multidisciplinary meeting between the gynecology and radiology specialists. Ultimately, it was concluded that the MRI was most consistent with uterine didelphys with a superior muscular septum and inferior fibrous division between two cervices - a different characterization from the initial MRI assessment. Both the minimally invasive gynecology (MIGS) and reproductive endocrinology and infertility (REI) specialists recommended an exam under anesthesia (EUA) and hysteroscopy for further evaluation and characterization of her anatomic variant, given the conflicting data to this point by imaging alone. 

**Figure 3:**
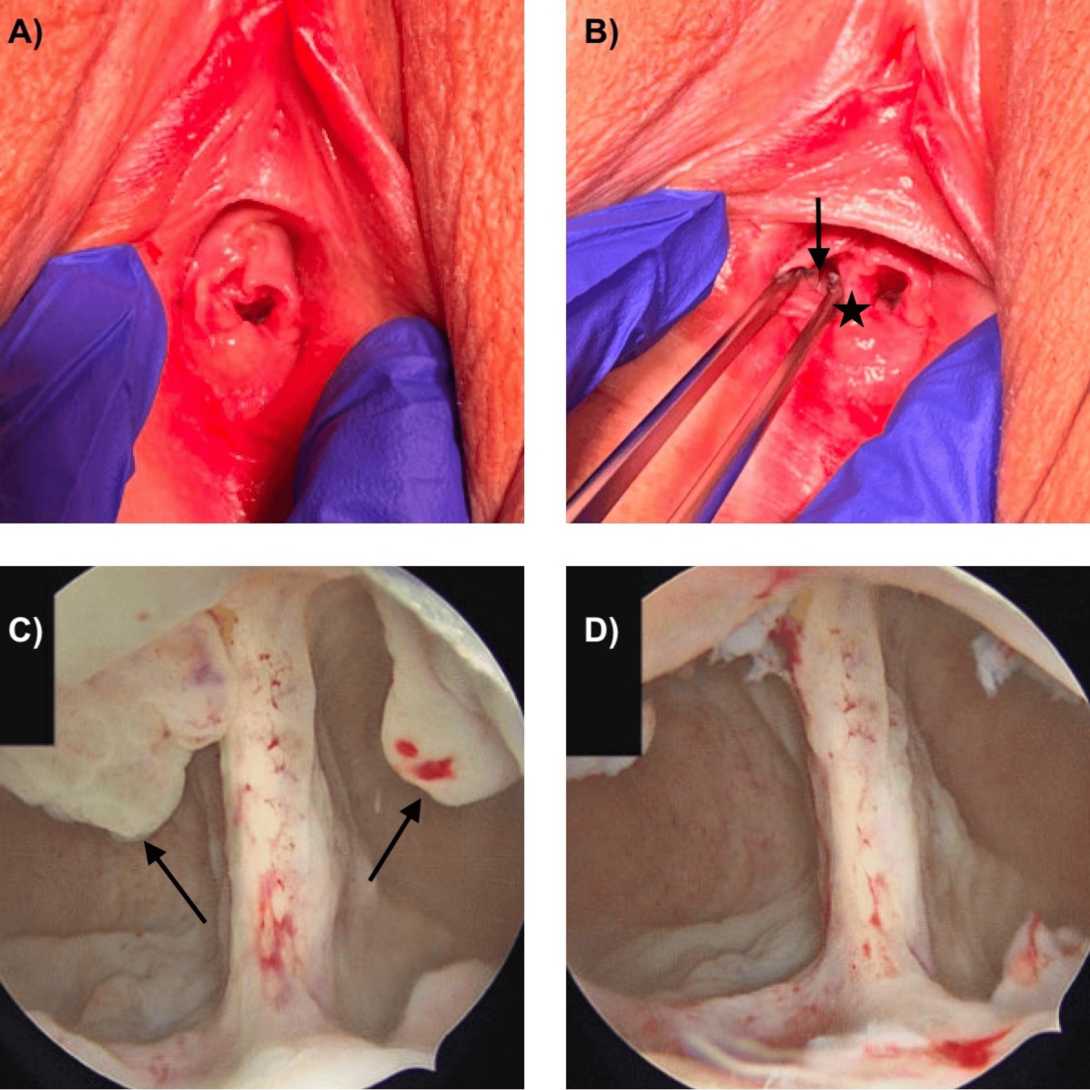
Operative findings Exam under anesthesia (A, B) demonstrating two hemi-vaginas with a longitudinal septum to the hymen (indicated by a star). The rudimentary right hemi-vagina is indicated with an arrow. (C) Hysteroscopy demonstrating two uterine cavities with communication in the lower uterine segment. Endometrial polyps in both cavities are indicated with an arrow; the polyps were removed at the time of hysteroscopy (D).

During the EUA (Figure 3), the vaginal introitus was notable for a thick vaginal septum extending the full length of the vagina to the hymen, with two asymmetric hemi-vaginas - one of more typical caliber on the patient left and another a hypoplastic hemi-vagina on the patient right. Each hemi-vagina had a cervix, notably the left cervix appearing well developed, while the right cervix was smaller but otherwise anatomically normal. Upon hysteroscopy, two uterine horns were observed with a small communication between the two cavities, just above the level of the internal ostium of each cervix. During a thorough survey of the cavities, polypoid tissue was identified at the 12 o’clock position within each uterine horn. Both polyps were removed without complication using hysteroscopic graspers and scissors. Histologic assessment of both specimens was consistent with benign endometrial polyps. 

According to the 2021 ASRM MAC system [1], based on the imaging and exam findings, the patient’s Müllerian anomaly could be best described as a uterine didelphys with communication between endometrial cavities, cervix bicollis, and longitudinal vaginal septum. 

The findings of the EUA and hysteroscopy were discussed with REI and maternal-fetal medicine (MFM) specialists; septum resection was not recommended for fertility given the findings of a uterine didelphys. However, she could consider a resection of the vaginal septum to address her dyspareunia. The patient declined surgical resection of her vaginal septum. She went on to conceive spontaneously, with the pregnancy located in the right uterine horn. At the time of publication, pregnancy is ongoing in the third trimester without complication with a cephalic fetus in the right uterine horn. Given the findings of the hypoplastic right vagina and cervix with unresected longitudinal vaginal septum, MFM specialists recommended primary cesarean delivery. 

## Discussion

The anomaly reported here appears in the updated 2021 ASRM MAC, but it has not previously been described in the literature. In this case, the patient’s Müllerian anomaly was sequentially assessed using three different imaging modalities: 2D TVUS, MRI, and SIS with 3D reconstruction. Each of these imaging modalities accurately described certain features, but no single modality provided a completely accurate assessment of her anatomic variant, which was conclusively evaluated at the time of her EUA and hysteroscopy (Table 1). The precision of any imaging modality in characterizing an anomaly is dependent on the technique of the radiographic technician and interpreting physician. While the initial assessment of the MRI was interpreted as a septate uterus with a flat fundal contour, the second opinion MRI interpretation correctly identified a deep fundal cleft consistent with uterine didelphys, similar to the imaging findings suggested by 2D TVUS ultrasound and SIS with 3D reconstruction. Both MRI and 2D TVUS identified the two cervices, while the exam during SIS and SIS images only noted one cervix. As only one cervix could be visualized and cannulated during the SIS secondary to patient discomfort, the SIS was incomplete and the demonstration of a single cervix on the imaging was inaccurate. Of note, on examination under anesthesia, the hemi-right vagina could only be appreciated with the use of a pediatric speculum, so it is not surprising that a thorough pelvic exam was difficult at the time of SIS given patient discomfort. Neither the MRI nor the initial 2D TVUS identified the communication between the endometrial cavities, but this was identified on SIS. Ultimately, a secondary reading of the MRI was most concordant with the EUA and hysteroscopy findings but still contained inaccuracies compared to her true diagnosis. 

**Table 1:** Comparison of findings based on imaging modalities While the 2D transvaginal ultrasound and pelvic MRI could identify the uterine didelphys, only the saline infusion sonohysterogram identified the communication in the lower segment of the uterus. None of the imaging modalities were sensitive enough to distinguish the vaginal septum. (+): feature identified with corresponding modality; (-): feature not identified with corresponding modality; (+/-): equivocal finding with corresponding modality

Anatomic finding	2D transvaginal ultrasound	Pelvic MRI	Saline infusion sonohysterogram with 3D reconstruction	Exam under anesthesia and hysteroscopy
Uterine didelphys	+/-	+	-	+
Bicornuate uterus	+/-	-	+	-
Communication in the lower uterine segment	-	-	+	+
Cervix bicollis	+	+	-	+
Longitudinal vaginal septum	-	-	-	+

Establishing the correct classification of a Müllerian anomaly is crucial for determining whether possible surgical intervention is warranted for fertility benefit, as studies have found that surgical intervention can improve pregnancy and live birth rates for specific anomalies [8]. In addition, correct classification can help inform preconception and pregnancy risk counseling and aid in labor and delivery management. However, as is demonstrated in this case, establishing a definitive diagnosis may be time-intensive and may require the integration of data from multiple diagnostic tools. TVUS and hysterosalpingography are the imaging modalities initially used in the work-up for infertility, but MRI is currently considered the best method for the evaluation of Müllerian anomalies [6]. While it would be optimal to only use less expensive imaging modalities including 3D ultrasound and SIS for the evaluation of Müllerian anomalies [7], there are not yet large studies to compare the sensitivity and specificity of these imaging modalities to MRI. 

This case report demonstrates that while the MRI results were most similar to the patient’s true anatomic variant, the TVUS and SIS showed comparable findings. TVUS is reasonable for an initial assessment of Müllerian anomalies, as this imaging modality has been established to distinguish a septate uterus from a bicornuate uterus based on the angle between the two uterine cavities [9]. However, as Müllerian anomalies have been found to be associated with renal anomalies (most common being unilateral renal agenesis) [10,11], it is important to consider assessment of the kidneys and ureters in patients with Müllerian anomalies. This can be accomplished with either an additional renal-bladder ultrasound or abdominal MRI to assess for renal anomalies in all patients diagnosed with a uterine anomaly [11]. Ultimately, any suspected Müllerian anomaly will require more than a singular ultrasound for characterization; in this case, imaging was unable to fully characterize findings that required a dedicated physical exam and direct visualization with hysteroscopy. Based on the case presented, we posit that it is reasonable to perform a 2D TVUS for an initial work-up followed by an MRI. If needed, EUA with hysteroscopy can then be performed if there are indeterminate radiological findings. 

In this case report, communication in the lower uterine segment demonstrates some of the shared features amongst different Müllerian anomalies. Based on the 1988 classification criteria [12], a uterine didelphys does not have any communication between the two horns. The 1988 classification criteria also delineates that there is always communication between the horns in a bicornuate uterus. However, the newest classification system (2021 ASRM MAC system) expands on the variations of the anomalies, including the possibility of communication between the endometrial cavities in uterine didelphys [1]. Similarly, a bicornuate uterus may not necessarily have communication between the two horns, as in bicornuate bicollis. While the new classification proposes numerous types of Müllerian anomalies, there is limited data to inform their clinical significance, and not all anomalies in this classification system have been reported in the literature and may be theoretical. This case report is the first known publication of this specific anomaly that newly appears in the 2021 ASRM MAC (uterine didelphys with a communication of endometrial cavities in the lower uterine segment, two cervices, and a longitudinal vaginal septum). It is unclear how to perfectly incorporate the previous data on uterine anomalies and fertility outcomes for future clinical management, as existing data on fertility outcomes uses anomaly diagnoses that were made prior to the new classification system. Future data on fertility outcomes using the 2021 ASRM MAC system will be necessary to provide accurate counseling to patients with complex anomalies.

## Conclusions

Detailed here is the multimodal radiographic evaluation of a complex Müllerian anomaly that was ultimately diagnosed as a uterine didelphys with communication in the lower uterine segment and longitudinal vaginal septum. The anomaly was of sufficient complexity such that an accurate diagnosis was not made until a physical exam under anesthesia and hysteroscopic visualization clarified data from previous MRI and ultrasonographic imaging. Appropriately identifying a Müllerian anomaly can be useful in patients with infertility and for preconception counseling, as there are both fertility and obstetrics risks for certain Müllerian anomalies.

Consistent use of the revised 2021 ASRM MAC system when reporting reproductive outcomes in Müllerian anomalies will be essential for more clearly understanding the impact of a particular anomaly and for providing improved patient counseling regarding fertility and obstetric care. Due to the nuances within various Müllerian anomalies, multiple imaging modalities may be necessary for the full characterization of uterine anomalies that may present with concomitant renal anomalies. 
